# Multivariate meta-analysis of proteomics data from human prostate and colon tumours

**DOI:** 10.1186/1471-2105-11-468

**Published:** 2010-09-17

**Authors:** Lina Hultin Rosenberg, Bo Franzén, Gert Auer, Janne Lehtiö, Jenny Forshed

**Affiliations:** 1 Clinical Proteomics, Department of Oncology-Pathology, Karolinska Institute/Karolinska University Hospital, Stockholm, Sweden; 2 Molecular Pharmacology, AstraZeneca R&D Södertälje, Sweden; 3 Department of Oncology-Pathology, Karolinska Institute/Karolinska University Hospital, Stockholm, Sweden

## Abstract

**Background:**

There is a vast need to find clinically applicable protein biomarkers as support in cancer diagnosis and tumour classification. In proteomics research, a number of methods can be used to obtain systemic information on protein and pathway level on cells and tissues. One fundamental tool in analysing protein expression has been two-dimensional gel electrophoresis (2DE). Several cancer 2DE studies have reported partially redundant lists of differently expressed proteins. To be able to further extract valuable information from existing 2DE data, the power of a multivariate meta-analysis will be evaluated in this work.

**Results:**

We here demonstrate a multivariate meta-analysis of 2DE proteomics data from human prostate and colon tumours. We developed a bioinformatic workflow for identifying common patterns over two tumour types. This included dealing with pre-processing of data and handling of missing values followed by the development of a multivariate Partial Least Squares (PLS) model for prediction and variable selection. The variable selection was based on the variables performance in the PLS model in combination with stability in the validation. The PLS model development and variable selection was rigorously evaluated using a double cross-validation scheme. The most stable variables from a bootstrap validation gave a mean prediction success of 93% when predicting left out test sets on models discriminating between normal and tumour tissue, common for the two tumour types. The analysis conducted in this study identified 14 proteins with a common trend between the tumour types prostate and colon, i.e. the same expression profile between normal and tumour samples.

**Conclusions:**

The workflow for meta-analysis developed in this study enabled the finding of a common protein profile for two malign tumour types, which was not possible to identify when analysing the data sets separately.

## Background

Emerging molecular pictures of cancer makes it evident that cancer portrays a group of diseases with numerous mutations and effected cellular pathways cross many different tumour types. The same permuted pathways drive tumour growth and metastasis in various cancer types; however some of the molecular features are tissue and tumour type specific. The key to personalized cancer therapy is the ability to classify and characterize tumours by molecular features to then be able to select the therapy [1]. Proteome analysis can provide valuable phenotypic information of tumour tissue on the molecular level. Proteomics, the global analysis of a proteome, has contributed to the understanding of global protein changes in cells [2].

Two-dimensional gel electrophoresis (2DE) has been, and still remains, a fundamental tool in expression proteomics. Several of the 2DE studies published over the years have reported lists of differently expressed proteins that regardless of experiment, tissue and species to a large extent overlap [3]. To be able to further extract valuable information from existing 2DE data, meta-analysis in combination with multivariate methods will be explored in this work. A multivariate approach allows studying protein patterns rather than one protein at a time. A meta-analysis combines the data from several studies and can for example be used to study more general protein patterns over several different tumour types. Merging data from several tumour types enables the investigation of several clinical issues not possible to answer based on a single data set. I.e. general molecular changes related to tumour progression such as proteins and protein patterns manifesting highly metastatic tumours, but also biomarkers specific for a certain tumour type as well as those biomarkers that reveal tissue of origin of metastatic disease. Specific biomarkers thus have the potential to help provide a more certain tumour diagnosis in cases where patients have a tumour for which the site of origin remains uncertain after the initial diagnose. Knowing the tumour origin enables more appropriate cancer treatment by using therapies that target specific tissues. General tumour biomarkers on the other hand can be used to better understand tumour biology and address common issues such as malignity, severity, survival rates and risk of metastasis. Identifying one common cancer-type independent protein signature is important to better understand cancer pathogenesis and ultimately also to improve diagnostics and therapeutics. A meta-analysis also allows distinguishing the common proteins playing a crucial role in oncogenic processes from those that are differently expressed only in certain tumour types.

Prerequisites to perform a valid meta-analysis of proteomics data are standardised methods from sample preparation to proteomics data generation. Over the last ten years a large amount of human tumours have been analyzed using 2DE standardised operation procedure at the Department of Oncology-Pathology (Karolinska Institute). The tumours have been handled using a standardized protocol, reproducible commercial IPG strips and standard gel electrophoresis as well as staining protocol have been applied and the expression data have been analyzed using the same software; PDQuest™, Bio-Rad Laboratories [4]. So far, each tumour type has been treated as a separate data set and the results have been published as individual publications [5,6]. Only a few other publications have looked at data from more than one tumour type [7,8]. These papers show common features between tumour types and demonstrates the potential of classifying tumours based on proteome changes. The aim of this study is to perform a meta-analysis of existing 2DE data and couple together data from colon and prostate human tumours. By establishing an expression database containing expression levels of the detected protein spots for 73 samples, a larger and hence better material for the selection of biomarkers is generated. The standardized handling of samples in this study and the following expression analysis using the same software forms the basis for a unique possibility. We can now take charge of data from previous studies and analyze the raw spot data on a higher level in a meta-analysis.

To answer the biological questions at issue, to identify proteins distinguishing between normal and tumour samples independent of tissue origin, a bioinformatic workflow was developed, dealing with pre-processing of data and handling of missing values followed by the development of a multivariate model for variable selection. Proteomics generates measurements of thousands of proteins in parallel, placing a large demand on the statistical method used to analyze it. Various methods for selecting variables have previously been used in proteomics biomarker studies, both univariate methods investigating the behaviour of one variable at a time, and multivariate methods looking at the behaviour of several variables simultaneously [9,10]. The information held in a large scale proteomics data set is complex, changes in expression by one protein may not be significant alone but rather a profile of protein expression changes can better describe the complex biology. The strength of multivariate methods is the possibility to define combinations of proteins that maximizes the model predictive ability. The use of multivariate methods such as Partial Least Squares (PLS) [11,12], where the expression of several genes or proteins are studied simultaneously, is increasing and has earlier shown to be powerful in tumour classification and biomarker discovery [13-17]. In this study, PLS was utilized to select the most important proteins for distinguishing between normal and tumour samples.

This work aims to show how to use existing 2DE data to perform analysis of tumour and normal tissue cross different tumour types by multivariate meta-analysis towards molecular based classification and characterization of tumour diseases.

## Methods

### Samples and 2D gel electrophoresis

The multivariate meta-analysis performed in this study was based on 39 prostate samples and 34 colon samples, collected and prepared as described in [5] and [6]. The tissue samples analysed by 2DE were from malign tissue as well as from corresponding benign tissue. The prostate data set contains 10 normal samples and 29 tumour samples. The colon data set contains 13 normal samples and 14 tumour samples, as well as 7 metastasis samples.

Two-dimensional gel electrophoresis was performed as described in references [18] and [6]. Briefly, the proteins were separated in the first dimension of isoelectric focusing using precast immobilized pH-gradient (IPG) strips with a pH 4-7 linear gradient (Bio-Rad Laboratories). The second dimension was performed in an Iso-Dalt tank (Hoefer, San Fransisco, CA USA) using 10-13% linear gradient SDS/PAGE gels. The gels were stained with silver nitrate and scanned using a flatbed scanner GS-710 (Bio-Rad Laboratories).

### Establishment of an expression database

The 2DE images from the prostate and colon sample sets were analyzed using the PDQuest software (version 7.0 and 7.3) [4]. The two sample sets were first analysed individually in the software. The software detects spots on every single gel image in the data set separately. The gel image containing the most spots is then selected as template (master) and all spots of the remaining gel images are matched onto it. The matched gels in the two data set form the "lower level" match sets. The prostate match set consisted of 39 samples and 1264 spots while the colon match set consisted of 34 samples and 1935 spots. Each spot in the match set was given a unique database identification number (SSP). PDQuest quantifies valid individual spots as parts per million of the total integrated optical density in the gel. The spot quantity corresponds to the amount of protein in the actual spot in the gel.

**Figure 1:**
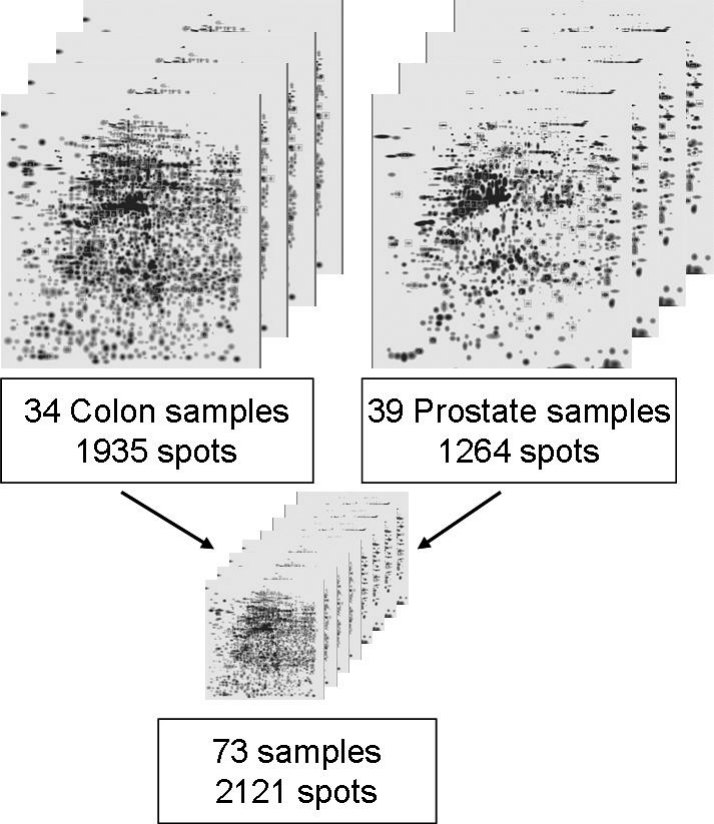
**Expression database**. 2-D gel photos from the two match sets A and B representing the samples and spots from the colon (A) and prostate (B) data sets. The match sets includes both different malign samples and the corresponding benign samples. In the higher level expression database (C) both match sets are linked together so that information from all the tissue types can be analyzed simultaneously.

The "higher level" matching between the prostate and colon "lower level" match sets was done in PDQuest (version 8.0.1). Using "higher level" matching the masters from the "lower level" match sets were matched to each other and thereby linked all the gels in the two data sets together. The resulting "higher level" match set consisted of 73 samples and 2121 spots in total (Figure 1). The intensity levels of the 2121 spots in all 73 gels were exported to perform statistical analysis in R [19].

There is of course a risk of mismatches in the matching procedure. That is, one spot from one protein is by accident matched to a spot from another protein, or alternatively not matched to any other spot. The risk of mismatches is largest in areas where clusters of spots appear. The automatic matching performed by the PDQuest software was therefore followed by manual inspection and improvement of the matches. This was done with special care in crowded gel-areas.

In addition to the true biological missing values, the image mapping approach in PDQuest can cause missing values. If a spot is missing in one of the lower level match sets, it will be replaced by the software with an estimate of the minimum detectable spot value. A spot missing in the higher level match set is replaced by zero. Approximately 59% of all data points in the higher level match set were missing. 30% of the missing data points are missing between data sets; that is they are completely missing in one of the two data sets; and 29% are missing within the data sets. One significant contributing factor to the large number of missing values is the very different amount of detected spots/proteins in the two data sets, 1935 and 1264 in colon and prostate respectively, resulting in many missing spots in the prostate data set.

### Data distribution and quality

Various diagnostic plots were used to assess the distribution and quality of the data and thereby judge weather any outliers were present in the data set and if any normalization or standardization was needed. The intensity values were log2 transformed to bring high intensity values together and stretch low intensity values to achieve a more symmetric data distribution. The density plot of log2 intensities (Additional file 1, Figure S1) revealed a rather homogenous data set with similar distributions of detected values for the different samples. The distribution of intensities is bimodal because of the missing values, which are given a low value by the software, different from the actual detected values. The intensity distributions for the different samples were further compared in a box plot (Additional file 1, Figure S2), excluding missing values. The plot revealed differences in median both between gels and between the tissue types, although not that pronounced considering the very different tissues. Principal Component Analysis [20] (PCA) was used for looking at trends and groups in the data. The PCA scores plot (Additional file 1, Figure S2) did not indicate any obvious outliers and also pointed out that the largest variation in the data is the origin of the samples. We tried to minimize the "batch effect" between the prostate and colon data sets seen in the PCA plot by normalization. Three different normalization methods were evaluated; normalization to equal total spot intensity between gels ("total intensity"), normalization to zero median intensity between gels ("gel median") and quantile normalization [21]. The normalizations were based on only non-missing data. Gel median and quantile normalization resulted in a more homogenous data distribution and removed the gel effect, although the "batch effect" seen in the PCA plot was unchanged for the normalized data (see Additional file 1, Figure S2).

Based on these observations, the decision was made not to perform any normalization of the data. The rationale behind this decision is that the "batch effect" still remained regardless of normalization method. Also, choosing the wrong normalization method gives a risk of introducing false differences and cancel out true differences between normal and tumour samples.

### Filtering of missing values

The alignment of the 2D gels in this study is a challenge because of the different origins of the analysed tissues. The differences in protein composition between samples give many missing values. A missing value can have several origins. It can be that the corresponding protein is absent in the sample, or the intensity was too low to be discriminated from the background level, or due to a problem of spot to spot matching (despite an extensive manual data curation). The amount of missing data can affect the multivariate analysis and which variables that will be selected. Although PLS can handle a small amount of missing values (5-20%), too many missing values can distort the multivariate analysis [22]. Thus, a filter had to be applied to remove some of the spots with a large amount of missing values before any further statistical analysis. Since the objective of this study is to compare cancer proteomes cross tumour types to reveal proteins connected to common tumour processes, the filter was based on the fraction of present values in both the prostate and colon gels. Using only the spots present in all gels in this study would result in only 60 spots, which is a great loss of data and a possible loss of interesting spots. Different fractions were investigated and the filtered data sets were studied by PCA to reveal how the filtering affected the grouping of the data.

### Estimation of missing values

Despite the exclusion of spots described above, the data set still contains missing data that has to be estimated with values. Many methods for handling missing values have been evaluated on microarray data [23], a field were the missing value problem is well known. Simple approaches are to impute the missing value by the average of the present values for the spot across all samples or to impute missing values by some minimal intensity value. Alternatively, local similarities in the data can be used to impute missing values, as in the k-nearest-neighbour imputing (KNN) [23]. Several of the methods for imputing missing values were recently evaluated on gel-based proteomics data [24]. The study revealed, in agreement with earlier literature, that KNN outperformed row mean and minimal value when applied to spots with low number of missing values (max 25%). For spots with high number of missing values (>25%) there was no effective method for imputing. The imputing methods also rely on different assumptions for the missing values, the KNN method for example assumes random occurrence of missing values and other spots having similar expression profile.

The distribution of the missing values over the whole data set as well as the distribution of missing values over the tissue types was investigated for this data set (Additional file 1, Figure S3). As seen in the plot, the distribution of missing values is not random for this data set; the amount of missing data is dependent on tissue type. Because of the very different tissues analysed, many of the missing values are also expected to be biological differences. Several of the assumptions mentioned are hence generally not applicable in this 2DE data study. Therefore, the expression patterns for present proteins provides a poor basis for estimation of missing values in the current study [25]. The values missing because of low or lacking expression of the corresponding protein are best exchanged by some low value. A value missing because of a mismatch is more difficult to estimate. The true protein level can be both low and high. Estimating it with a mean value will influence the model least, and hence cause as little false effects as possible [22]. Since the true state of the missing values is not known, and a manual inspection of 2121 spots in 73 gels is not feasible, two basic methods for estimating the missing values were tried out in this study. At first, all the missing values were exchanged by the mean value over samples (from all tissues) for the spot. Secondly, all the missing values were exchanged by the value for the sample at the 10% lowest value. The two methods for estimating the missing values were run in parallel through out the multivariate analysis and evaluated at the end of the project.

### Multivariate analysis of data - PLS

The number of predictor variables (2121) in this data set greatly exceeds the number of observations (73). PCA and PLS are multivariate projection methods that can handle high dimensionality of the data, as well as the presence of a large amount of biological noise [15]. PCA is an unsupervised method, useful for getting an unbiased overview of the data as well as to detect trends and outliers. PCA reduces the dimensionality of the data set, **X**, by introducing a new set of variables, latent variables, which maximize the variance of a linear combination of the predictor variables. To be able to integrate information about the response variable **y **into the model, a supervised method like PLS has to be used. PLS models are based on finding the latent variables (also called PLS components) in the data that maximize the covariance between the response variable, **y**, and a linear combination of the predictor variables [12]. PLS is suitable for analyzing data where the number of variables greatly exceeds the number of observations. Further, the PLS model relies on linear algebra which gives transparent models and a straight forward interpretation of the variable's influence on the model and on the classes in data. In this study, PLS discriminant analysis (PLS-DA) [15,26] was utilized to select the optimal set of protein spots for discriminating between normal and tumour samples. For the multivariate analysis purpose the data was divided in two classes: *normal *consisting of colon normal (13 samples) and prostate normal (10 samples) and *tumour *consisting of colon tumour (14 samples) and colon metastasis (7 samples) and prostate tumour (29 samples). Because of the malignant property of the metastasis samples (verified by the PCA plot where the metastasis samples group with tumour samples), they were placed together with the tumour samples in this study. The response variable **y **is thus a binary vector of classes coded as 0 for normal samples and 1 for tumour samples.

### Variable selection and validation of PLS model

Although PLS can handle a large number of predictor variables (thousands), only a subset of the variables are expected to be of biological interest for this study. There are several approaches for reducing the number of variables [9,10], in this project a wrapper method was used where the variable selection is connected to the variables performance in the PLS model. The variables were ranked by the PLS dependent Variable Importance on Projection (VIP) score [27], a summary of the importance of an **X **variable for both **X **and **y**. In a backward elimination strategy starting with the full set of variables, the number of variables was then decreased by 5% in each step, excluding the lowest ranked variables.

**Figure 2:**
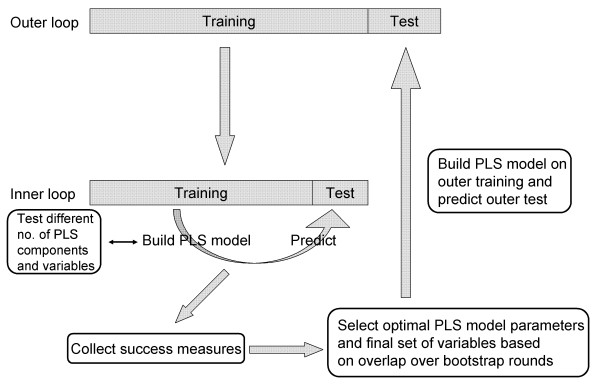
**Double cross-validation scheme**. In the inner loop, PLS model parameters and variables are estimated based on a bootstrap cross-validation. Based on performance of the PLS models and stability of variables over bootstrap rounds, the optimal parameters and final set of variables are selected. Model performance of the optimized parameters and selected variables are then evaluated on the held-out test set in the outer loop. The outer loop is repeated within a 5-fold cross-validation procedure.

To avoid the risk of over-fitting the model to the data and to be able to evaluate the model performance on a held-out test set, different kind of cross-validation can be used. The modelling procedure in this study was performed in two nested cross-validation loops, an inner loop to optimize model parameters and select variables and an outer loop to measure the optimized model performance on a held-out test set [28,29]. A schematic view of the double cross-validation scheme is shown in Figure 2. In the outer loop, a full cross-validation was used, by randomly dividing the data in five parts once, making sure each subset contained at least one sample from each of the classes normal and tumour. One part in turn is set aside as a test set (20%) and the rest was used to optimise the PLS model and select variables. In the inner loop, a bootstrap cross-validation [30] was used. The data was then randomly divided in a 80% training and a 20% test data set 500 times (see Results), and the mean predictive performance were calculated. The bootstrap validation was chosen due to the few and diverse samples in this data set, to establish a stable estimate of prediction power. The minimum number of bootstrap rounds needed to give stable success measures was investigated. The different number of PLS components and variables were evaluated by calculating the success measures when applying the model to the test sets and the optimal PLS parameter settings was decided as the minimal number of PLS components and variables still giving a good predictive power. The final set of variables was selected based on stable variables from the bootstrap validation in the inner loop; variables selected in at least 50% of bootstrap rounds. The final set of variables and the optimal PLS model was used to predict the test set excluded in the outer loop. The cross-validation in the outer loop was repeated for all five parts and the resulting success measures and variable lists were compared.

The success measure used to evaluate the PLS models was the geometric mean of sensitivity and specificity, a measure not influenced by the size of the classes [31,32]. The success measures for the original model, using VIP selected variables, were compared to results based on the same number of randomly drawn variables.

The R code (Additional file 2) written for the multivariate analysis is available from the first author upon request.

## Results

### Handling of missing values

**Figure 3:**
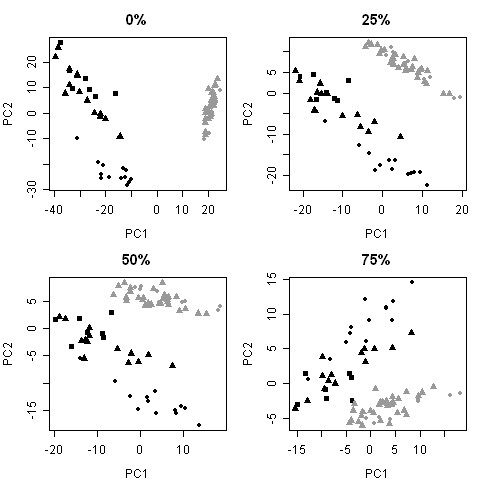
**PCA scores plots for filtered data sets using different criteria**. The filtering is based on fraction of present spots in both tissue types; spots present in at least 0, 25, 50 and 75% of both prostate and colon samples are included. Black circles are colon normal samples, black triangles colon tumour and black squares colon metastasis samples. Grey circles are prostate normal samples and grey triangles are prostate tumour samples.

The matching of gels between the two tissue types is a very difficult task because of the inheritably different protein expression patterns. Missing spots can be a result of mistakes in the matching procedure as well as truly missing proteins. The amount of missing values strongly affects the multivariate analysis and which variables that are picked up as important. Pre-filtering of spots was hence necessary to remove some of the uncertain spots with missing values in many of the samples. The filtering of spots was based on fraction of present values over both prostate and colon samples. The criteria of 25%, 50% and 75% present values in both prostate and colon samples were investigated. The filtering of missing values had a strong impact on the grouping of the data which is visualized in the PCA scores plots in Figure 3. When including all 2121 spots the largest variation is between the data sets (prostate and colon) - called the "batch effect", while the second largest variation is the spread within the data sets. This is most likely a result of the many missing values, spots with missing values in one of the data sets but not in the other contributes to a large variation in the data. When excluding spots missing in one data set (using 25%, 50% or 75% present as filter criteria) the largest variation is shifted towards the spread within the data sets. This filtering thus decreases the "batch effect" seen in the PCA scores plot (Figure 3) caused by integrating the two disparate data sets generated by two different prior studies. Based on these results, the decision was made to exclude any spots that are totally missing from either prostate or colon data set. The minimum level of present values was set to 25%, not to exclude any spots present in only the smallest subclass of the data (10/39 = 26%). This filter criterion implies that a spot has to be present in at least 25% of both prostate samples and colon samples simultaneously to be included. After applying the filter the data set consisted of 731 spots which formed the basis for the continued multivariate analysis.

Once the spots with a large amount of missing data were filtered out the remaining missing data points had to be estimated with values. The missing values were either exchanged by the mean value of the spot (from now on called "row mean") or by the 10% lowest value for the spot (called "ten lowest"). The two different methods were run in parallel through out the following analysis and evaluated first at the end of the project.

### Multivariate analysis of data

**Figure 4:**
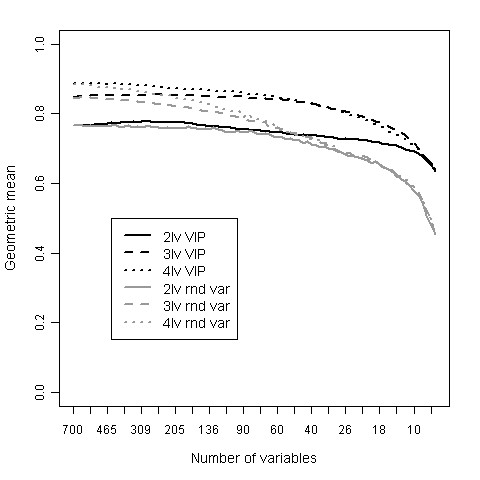
**Prediction success for different PLS model parameter settings**. Average of geometric mean of sensitivity and specificity over 500 bootstrap rounds in inner loop using different number of PLS components and different number of variables. Results for missing value method "ten lowest". Black lines are results for the VIP selected variables and grey lines are the corresponding results for the same number of randomly picked variables. Solid lines represent two PLS components, dashed three PLS components and dotted four PLS components.

500 bootstrap rounds in the inner loop of validation were found to give stable success measures (Additional file 1, Figure S4). The mean success rate over the 500 bootstrap rounds for the missing value method "ten lowest" can be seen in Figure 4. Results for the same number of random variables are presented in the same plot. The optimal number of variables in the PLS model is a trade-off. The number of selected variables should be small enough to enable further validation of the proteins using a more targeted protein analysis method for measuring the expression levels in clinical samples. At the same time, the number of variables has to be large enough to achieve a good predictive PLS model. When decreasing the number of variables in the PLS model, the VIP selected variables outperforms the randomly picked variables. As seen in figure 4, including more than around 50 variables does not give any significant improvement of the success rate and with fewer variables the success rate starts decreasing rapidly.

Regarding the optimal number of PLS components, for more than 60 variables four PLS components gives a slightly better result than three. For models with less than 60 variables, three PLS components seem to be enough. Hence, three PLS components is enough to describe the data with 60 or less variables and the fourth PLS component is most likely just describing noise. Thus, three PLS components and 50 variables were selected as optimal PLS parameter settings for the missing value method "ten lowest". Using the same reasoning for the missing value method "row mean" gave a PLS model with three PLS components and 60 variables (Additional file 1, Figure S5).

The final selection of variables was based on stability over the bootstrap validation rounds from the inner loop. Variables selected in at least 50% of the 500 bootstrap rounds were identified for each of the five cross-validation sets in the outer loop. This five lists of variables (named "stable variables" from now on) is thought to represent variables generally good for predicting the classes and not specific for certain subsets of the data. Despite such different tissues in the data, there were around 40 variables (from the lists of 50 variables) that were selected in at least 50% of the bootstrap rounds for the missing value method "ten lowest". For missing value method "row mean" there were around 50 variables (from the lists of 60 variables) that were selected in at least 50% of the bootstrap rounds. Those stable variables were together with the optimised PLS model applied to predict the held-out test sets in the outer loop. The resulting prediction success measures are presented in Table 1. The missing value method "ten lowest" gave a better prediction performance than the missing value method "row mean". The average geometric mean of sensitivity and specificity over five cross-validation rounds was 0.93 (± 0.06) and 0.73 (± 0.16) for "ten lowest" and "row mean" respectively.

**Table 1:** Prediction success measures for held out test-sets

	Row mean			Ten lowest		
	
CV round	Sensitivity	Specificity	G mean*	Sensitivity	Specificity	G mean*
1	0.90	0.40	0.60	1.00	0.83	0.91
2	1.00	0.75	0.87	1.00	1.00	1.00
3	1.00	0.38	0.61	1.00	1.00	1.00
4	0.88	1.00	0.94	1.00	0.8	0.89
5	0.96	0.40	0.63	1.00	0.75	0.87
Average	0.96	0.59	0.73	1.00	0.88	0.93
Std	0.06	0.28	0.16	0	0.12	0.06

*geometric mean of sensitivity and specificity

Prediction success measures for the two missing value methods "row mean" and "ten lowest" for held-out test sets in the outer loop cross-validations using optimised PLS model and stable variables.

### Further analysis of stable variables

**Figure 5:**
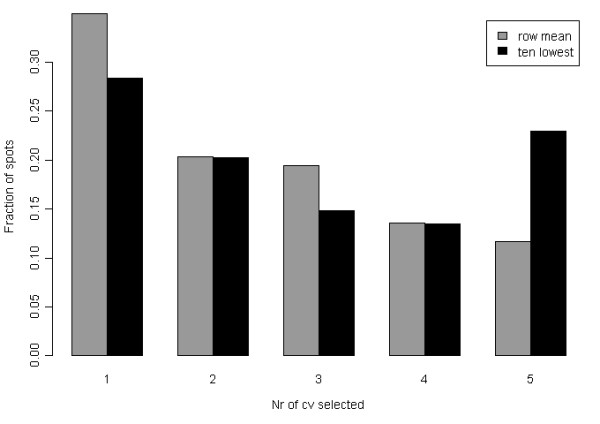
**Overlap of variables over cross-validation rounds**. Overlap of stable variables over outer loop cross-validation rounds for the two methods of handling missing values. Black bars represent the "ten lowest" and grey bars represent the "row mean" method.

**Figure 6:**
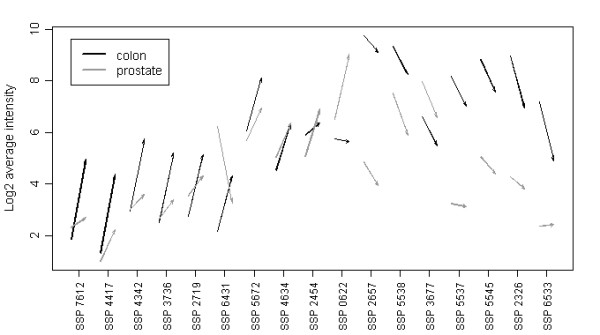
**Expression profiles for overlapping stable variables**. Average expression profiles for the 17 stable variables selected in all five cross-validation rounds for the missing value method "ten lowest". The arrows indicate the differences in expression levels going from normal to tumour samples, starting in average expression level for normal samples and ending in average expression level for tumour samples. Black arrows represent up and down regulations in the colon samples and grey arrows represent the regulations in prostate samples. The SSP XXX are spot identification numbers given by the PDQuest software.

After assessing the optimal number of PLS components and number of variables based on bootstrap cross-validation, the full data set is used to further point out the most important variables. The five lists of stable variables from outer loop cross-validation rounds were merged and redundancy removed. The resulting lists contained 103 variables for the missing value method "row mean" and 74 variables for the missing value method "ten lowest". The amount of overlap over cross-validation rounds for the lists is presented in Figure 5. Using the missing value method "ten lowest" results in a larger overlap of stable variables over all five cross-validation rounds than using "row mean", 17 variables are present in all the five lists using "ten lowest" while the corresponding number for "row mean" is 12. The overlapping variables, 17 and 12 respectively, hence represents variables with a strong profile that are selected independently of which samples that are used as training samples. The prediction performance using only the 17 overlapping variables for the missing value method "ten lowest" were compared to the performance using all stable variables (one list for each cross-validation set) as well as using 17 randomly selected variables from the stable ones (Table 2). Despite the much smaller number, the overlapping 17 variables gives as good prediction performance as the around 40 stable variables. This is not seen for the same number (17) of randomly selected variables. The average expression profile for the two tumour types for the 17 overlapping variables from the missing value method "ten lowest" are seen in Figure 6. As seen in the figure there are differences between the expression levels between the two tumour types, colon and prostate. It can also be seen that the differential expression direction for 14 of the 17 variables agrees. Eight of the variables with an up-regulation in expression between normal and tumour samples agree between colon and prostate and six of the variables with a down-regulation in expression agree.

**Table 2:** Prediction success measures for overlapping variables

	Sensitivity		Specificity		Geometric mean*	
	
Variables	Average	Std	Average	Std	Average	Std
~40 Stable	1.00	0.00	0.88	0.12	0.93	0.06
17 Overlap	1.00	0.00	0.89	0.10	0.94	0.05
17 Random	0.94	0.09	0.79	0.14	0.86	0.10

*geometric mean of sensitivity and specificity

Average prediction success measures for variables selected using the missing value method "ten lowest". A PLS model with a five fold cross-validation (according to Figure 2) was built for three sets of variables: ~40 stable variables (one list for each cross-validation round), 17 overlapping variables and 17 variables randomly selected from the stable ones.

The two different methods for handling missing values were evaluated also by studying the gel images of the spots selected in the PLS analysis. It was found that most of the missing values among those spots were results of truly missing spots on the gel, a result of a missing or low abundant protein in that sample. This finding implies that the method of exchanging the missing values with the 10% lowest value for the spot is the preferred method.

### Comparison of meta-model to individual models

**Figure 7:**
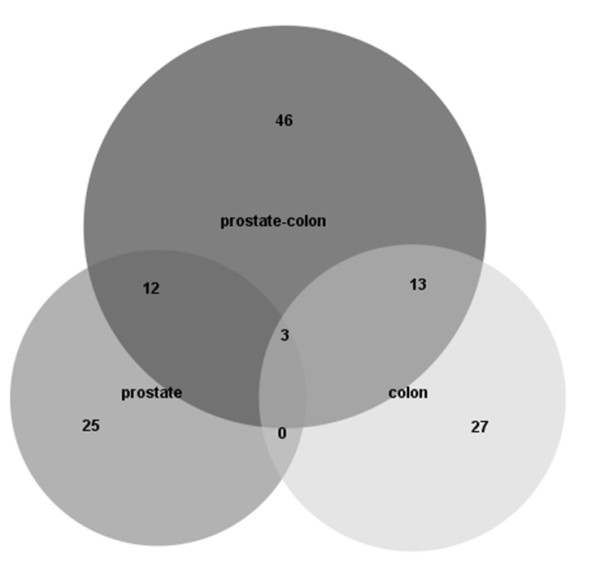
**Overlap of variables selected in different models**. Venn diagram showing overlap between stable variables selected using the meta-model (74 proteins) and individual prostate and colon models (40 and 43 proteins respectively). Results for the missing value method "ten lowest".

To compare the results from the combined colon-prostate model to individual colon and prostate models the above described PLS modelling was also applied to the colon and prostate data sets separately. The same classes, normal and tumour, was used in the modelling as well as the same set of 731 protein spots remaining from the missing value filtering. The resulting lists of variables selected from the five cross-validation rounds were compared. A Venn diagram showing the overlap between merged stable variables for the three models (prostate-colon, prostate and colon) can be seen in Figure 7. The figure reveals that most variables are unique to the models and few overlaps are identified, 46 variables selected in the meta-analysis of prostate and colon are not picked up in the individual models, 27 variables are unique to the colon model and 25 variables to the prostate model. Only three variables overlap between all three models, their average expression profile for the two tumour types colon and prostate are found in Additional file 1, Figure S6.

## Discussion

The aim of this project was to develop a data handling workflow to compare tumour proteomes cross different tumour types and use these methods to find proteins whose expression levels separate between normal and tumour samples independently of which tissue the samples come from. This was achieved by performing a meta-analysis. Gels from 2DE analysis of colon and prostate samples were matched and an expression database containing the intensity levels of detected spots in all samples was created. The tissues were very different and the corresponding gels were thereby very dissimilar and thus difficult to match. Many missing values existed in the merged data set, which effected the modelling result, thus the missing value problem had to be handled with care. Most methods for handling missing values rely on several assumptions not fully met for this data set. Furthermore, with the purpose of finding proteins with common expression patterns over the two tumour types, the analysis was restricted to those proteins that were expressed in both data sets. A novel workflow for handling this type of clinical proteomics data hence had to be developed.

The number of proteins that could be used in this meta-analysis were decreased because of the inherent biological difference of the tissue types studied, the rather small sample sizes of the individual studies and the variation due to experimental differences. At first, a filtering of spots with a large amount of missing values had to be used for excluding any spot completely missing from one of the datasets prostate and colon. A limitation of this ad-hoc procedure of filtering out the proteins missing in one dataset is of course that many potentially informative proteins may be excluded. The missing values remaining after applying the filter were exchanged by a value using two different approaches, which were run in parallel through out the multivariate analysis workflow. No data set fulfils all assumptions and there exist no perfect method for imputing missing values. Any choice made is more or less a compromise. The best is to treat biological missing values (that should be zero or a low intensity value) and technical missing values (with unknown levels) separately. Since the main cause of missing values in our data set was unknown, we had no assumption for guiding in choosing the best methodology for missing data estimation. The methods hence had to be evaluated at the end of the study, by comparing the variables selected and the prediction success.

This study utilized PLS-DA to build predictive models and to select variables important for separating between the classes normal and tumour no matter colon or prostate cancer. In any statistical modelling there is a risk of over-fitting the model to the data which in turn can lead to over-optimistic results and bad generalization of the model. Thus the validation of the model is a crucial step [9,10]. The key to protect against overly optimistic prediction performances and biases in variable selection is to avoid testing the model on the same data set that was used for model training and variable selection. In clinical proteomics, the sample numbers are often limited because the methods used for comprehensive proteomics studies are laborious and expensive. This was the case also in this study. With limited amount of data available, resampling techniques such as bootstrap- and cross-validation are useful to give an estimation of validity of the model. The optimal PLS model and the selected variables were evaluated in two nested loops of cross-validation in this study. The final set of variables was then selected based on several criteria: prediction performance, appropriate numbers of variables and stability of variables over bootstrap rounds. Despite such different tissues in the data sets, there were around 40 variables selected in at least 50% of the bootstrap rounds for the missing value method "ten lowest". From those variables, 17 were selected in all five cross-validation rounds, which reveal a strong profile for those variables. Studying the mean expression profiles in the data for the 17 proteins (Figure 6) it is also clear that there exist some common trends within the tissue types even though the expression levels are different between the tissue types.

The comparison of selected variables between the combined colon-prostate model and the individual colon and prostate models disclose that many of the variables are unique to the models. As many as 46 of the variables from the meta-analysis of colon-prostate did not show up in the individual models, while 27 and 25 variables were unique to the colon and prostate models respectively (Figure 7). This result supports the hypothesis that a meta-analysis adds extra value to the analysis of large scale cancer proteomics data and has the potential to identify proteins not found when analyzing the data sets in separate. The small number (three) of overlapping proteins between the individual colon and prostate models indicates that just comparing the signatures from the two data sets would probably result in very few common proteins. In the individual models, the more tissue specific proteins are picked up by the model and the common protein patterns are not as pronounced in comparison. By combining several tumour data sets we have identified protein profiles that can be used in addressing several clinical questions which are impossible to answer based on analysis of a single study. The 46 protein spots unique to the meta-analysis represent proteins whose expression levels discriminate between normal and tumour samples independent of tissue type in this study, i.e. a common protein profile for malign tumour types. The protein spots unique to the individual models on the other hand represent proteins that are specific for the certain tumour types prostate and colon. Since many cancer types share common characteristics, it is important to identify common protein signatures to better understand cancer biology. The finding of a common tumour profile can have potential to develop into useful biomarkers for diagnostics and ultimately to improve therapeutics. The results from this meta-analysis will also give clues to the complex biology of the human tumours prostate and colon. Further analysis will aim at identifying the selected proteins and gain more insight into their functions and interactions with each other and with other proteins in biological pathways. The study so far includes data from prostate and colon tumours and can function as a proof of concept of a meta-analysis workflow for 2DE data. The potential of including more tumour types is apparent.

## Conclusions

The workflow for meta-analysis developed in this study enabled the finding of a common protein profile for malign tumour types as well as tumour specific proteins. Despite the limited number and diverse samples included in this meta-analysis, resulting in a small number of overlapping proteins, variables that yielded a good predictive performance (93% geometric mean of sensitivity and specificity) were selected. For this study, when very different data sets were fused, a filter of non-overlapping proteins followed by a simple approach for estimating missing values proved to work well.

## Availability and requirements

Project name: Multivariate meta-analysis

Project home page: http://www.forshed.se/jenny/index.php?n=Research.SoftwareAmpCode

Operating system: Platform independent

Programming language: R

Other requirements: R 2.9.2 or newer, pls 2.0.1 or newer

License: GNU General Public License

## Authors' contributions

BF came up with the original idea of merging previously performed 2D studies. BF, JL, JF and GA conceived of the study. GA was responsible for the raw data collection. LHR planned and carried out the analysis of data, wrote the scripts used for analysis and drafted the manuscript. JF participated in the design of the study, came with input on the statistical analysis and helped to draft the manuscript. JL participated in the design and planning of the study and came with input on the manuscript. All authors read and approved the final manuscript.

## Supplementary Material

Additional file 1 **Supplementary figures S1-S6**. Word document containing supplementary Figures S1-S6. Click here for file

Additional file 2 **Source code**. A zip-file containing the R code for the project and example data. Click here for file
