# Supplementary material for: Multivariate meta-analysis of proteomics data from human prostate and colon tumours
Source: BMC Bioinformatics. 2010 Sep 17;11:468. doi: 10.1186/1471-2105-11-468 (PMC2949896; doi:10.1186/1471-2105-11-468)
Supplement: Additional file 1 — Supplementary figures S1-S6. Word document containing supplementary Figures S1-S6. [file 1471-2105-11-468-S1.DOC]

**Supplementary figures for “Multivariate meta-analysis of proteomics data from human prostate and colon tumours”**

**by Hultin Rosenberg L, Franzén B, Auer G, Lehtiö J and Forshed J**


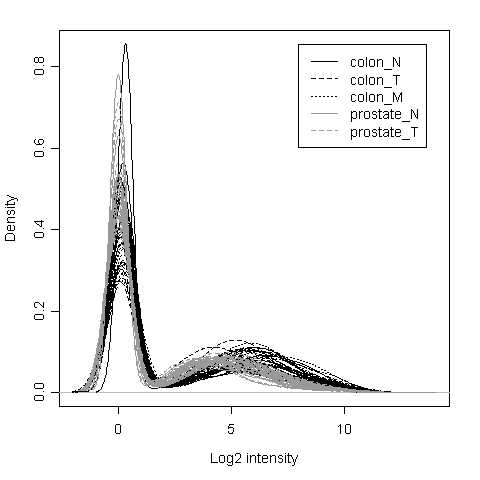


**Figure S1 - Distribution of the log2 intensity values for 2121 protein spots.** Each line represents one sample. Black solid lines are colon normal samples, black dashed lines colon tumour and black dotted lines colon metastasis samples. Grey solid lines are prostate normal samples and grey dashed lines are prostate tumour samples.


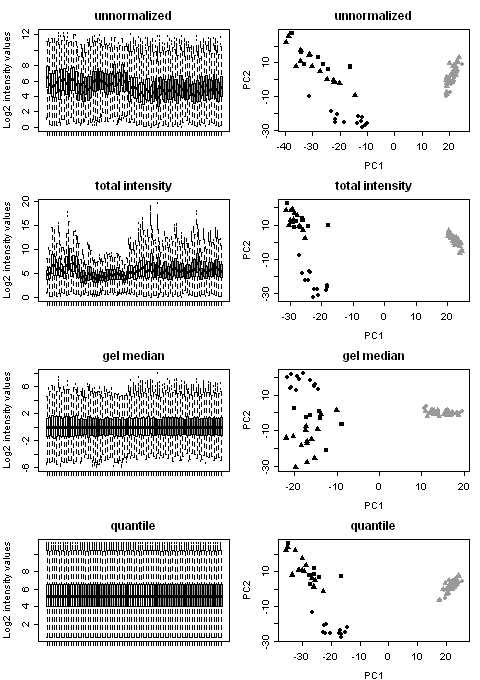


**Figure S2 – Box plots and PCA scores plots for different normalization methods.** The left panel shows box plots of log2 intensity values (non-missing values) before and after normalization. The order of tissue types from left to right is: colon normal (white boxes), colon tumour (dark grey boxes), colon metastasis (light grey boxes), prostate normal (white boxes) and prostate tumour (dark grey boxes). The right panel shows PCA scores plots before and after normalization. Black circles are colon normal samples, black triangles colon tumour and black squares colon metastasis samples. Grey circles are prostate normal samples and grey triangles are prostate tumour samples.


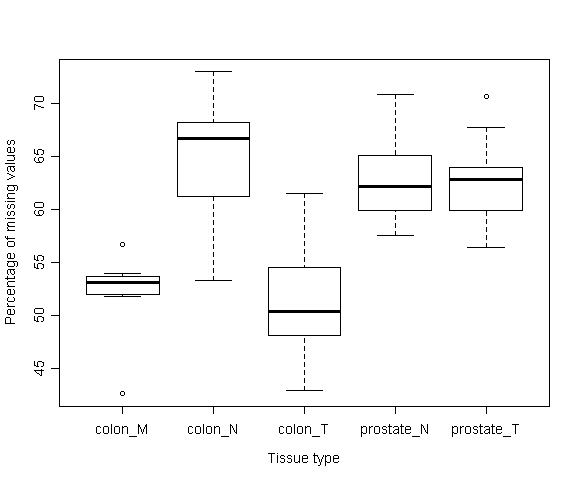


**Figure S3 – Box plot of percentage of missing values for the different tissue types.** Boxes show the interquartile range, (range between the 1^st^ and the 3^rd^ quartile) including 50% of the data, and median value. Whiskers extend to 1.5 times the interquartile range, any value beyond the extreme of the whiskers are outliers represented by circles in the figure. The tissue types are colon metastasis, colon normal, colon tumour, prostate normal and prostate tumour.


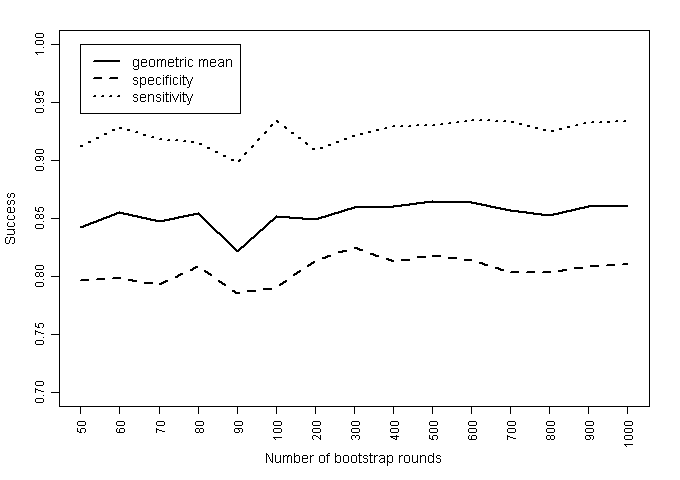


**
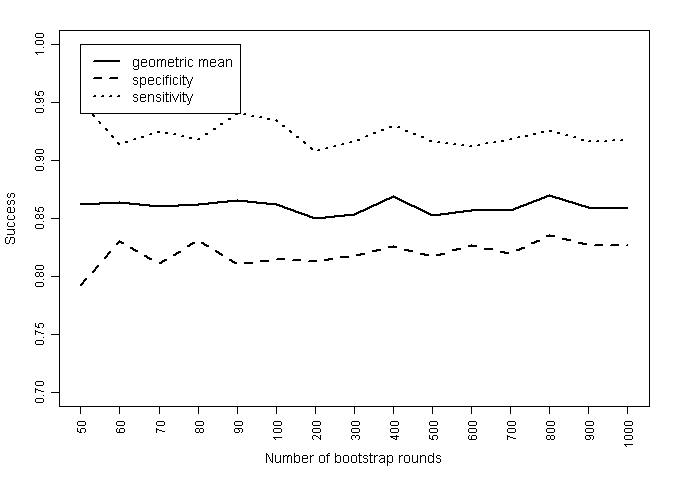
**

**Figure S4 - Success measures for different number of bootstrap rounds.** Investigation of number of bootstrap rounds necessary to reach stable success measures. The top panel represents results for missing value method “row mean” and the bottom panel for “ten lowest”.

**
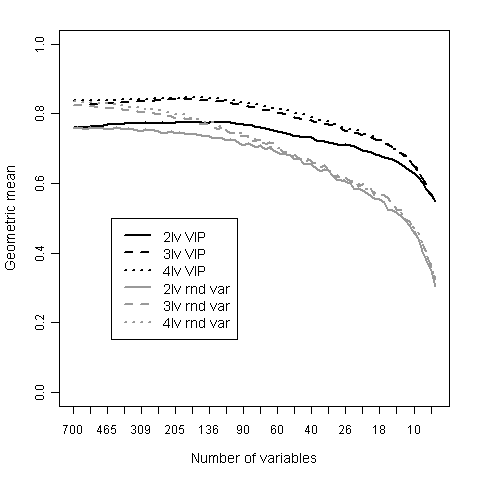
**

**Figure S5 - Prediction success for different PLS parameter settings.** Average of geometric mean over 500 bootstrap rounds using different number of PLS components (latent variables) and different number of variables. Results for missing value method “row mean”. Black lines are results for the VIP selected variables and grey lines are the corresponding results for randomly picked variables. Solid lines represent two PLS components, dashed three PLS components and dotted four PLS components.


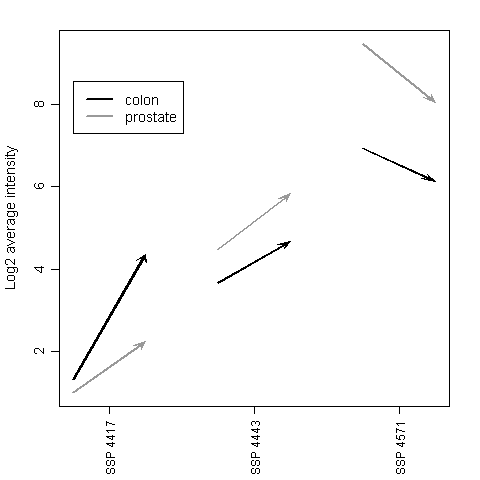


**Figure S6 - Expression profiles for overlapping stable variables.** Average expression profiles for the three stable variables overlapping between all three PLS models (prostate-colon, prostate and colon) for the missing value method “ten lowest”. The arrows indicate the differences in expression levels going from normal to tumour samples, starting in average expression level for normal samples and ending in average expression level for tumour samples. Black arrows represent up and down regulations in the colon samples and grey arrows represent the regulations in prostate samples.
